# Prophylaxis of Upper Airway Infections in a Patient with Partial IgA Deficiency: Concurrent Use of Sublingual Immunotherapy with Inactivated Whole-Cell Bacterial Extract and Der p1

**DOI:** 10.1155/2020/6313176

**Published:** 2020-08-24

**Authors:** Paula F. Aarestrup, Matheus F. Aarestrup, Beatriz J. V. Aarestrup, Fernando M. Aarestrup

**Affiliations:** ^1^Universidade Federal do Rio de Janeiro–UNIRIO, Rio de Janeiro, Brazil; ^2^Instituto de Ensino Superior Tancredo Neves–IPTAN, São João Del Rei, Minas Gerais, Brazil; ^3^Laboratory of Immunopathology and Experimental Pathology (CBR), Department of Morphology (ICB), Universidade Federal de Juiz de Fora, Juiz de Fora, Minas Gerais, Brazil; ^4^Chairman Laboratory of Immunopathology and Experimental Pathology (CBR), Universidade Federal de Juiz de Fora, Chief Allergy and Immunology Service, Faculdade de Ciências Médicas e da Saúde – SUPREMA e Hospital Maternidade Therezinha de Jesus, Juiz de Fora, Minas Gerais, Brazil

## Abstract

Selective IgA deficiency is the most common type of primary immunodeficiency, but there is not yet a specific effective treatment. The most prevalent clinical manifestations are infectious diseases of the respiratory system. We report herein the case of an 11-year-old female with selective IgA deficiency and recurring episodes of respiratory infections associated with rhinitis and asthma. We evaluated the efficacy of sublingual immunotherapy combined with inactivated whole-cell bacterial extract and Der p1-specific immunotherapy. After 18 months of clinical follow-up, we observed a significant reduction in the number of episodes of respiratory infections associated with control of atopic diseases. We also observed a 3-fold increase in serum IgA levels compared to treatment initiation. This case demonstrates the potential utility of the concurrent use of sublingual immunotherapy with inactivated whole-cell bacterial extract and Der p1 for successful control of allergy and infection in partial selective IgA deficiency.

## 1. Introduction

Selective IgA deficiency is the most common type of primary immunodeficiency in the world population [1], is particularly prevalent among people of Caucasian origin, and has no sex-based predilection. In general, a serum IgA level of less than 7 mg/dL (0.07 g/L) is considered as selective IgA deficiency since this concentration is the lowest detectable limit established by most of the laboratories. When the serum IgA level is higher than 7 mg/dL but two standard deviations below normal for age, the condition may be referred to as partial IgA deficiency. A definitive diagnosis should be made only after 4 years of age because undetectable or low serum levels may be observed until this age. Therefore, children under 4 years of age with suspected selective IgA deficiency should be periodically monitored to assess IgA serum levels, thereby confirming the initial diagnostic hypothesis [1, 2]. Occasionally, decreased serum IgG2 and/or IgG4 levels may also be observed in association with IgA deficiency, resulting in a more severe clinical impairment characterized by recurrent episodes of difficult-to-control bacterial infections.

Clinical manifestations of total or partial selective IgA deficiency vary from individual to individual. The most common clinical finding is an increased susceptibility to predominantly bacterial infections of the respiratory, gastrointestinal, and urogenital systems. Furthermore, allergic and autoimmune diseases are commonly observed in patients with selective IgA deficiency. Asthma is the main observed allergic disease. There is no evidence demonstrating an increased incidence of allergic rhinitis and atopic dermatitis in patients with IgA deficiency. However, rheumatoid arthritis, systemic lupus erythematosus, and other types of autoimmune diseases are more prevalent in patients with selective IgA deficiency than in the general population [2–4]. The possibility of patients with selective IgA deficiency showing anaphylactic reactions following intravenous administration of blood products is an extremely important clinical complication. In addition, Clark et al. [3] showed that 44% of patients with IgA deficiency have anti-IgA IgE antibodies.

Currently, there is no specific treatment for selective IgA deficiency. The therapeutic approach is individualized according to the clinical status of the patient. The use of antibiotic prophylaxis in patients with a history of recurrent respiratory infections may be established in specific cases of higher severity or characterized by high frequency of infectious episodes. The use of intravenous immunoglobulin is not indicated [1, 4]. Therefore, few treatment possibilities are currently available, particularly in patients with upper respiratory infections. Accordingly, patients who concurrently develop allergic diseases, especially asthma, have low quality of life and may develop severe clinical cases with difficult therapeutic management.

In the present study, we report the use of sublingual immunotherapy (SLIT) combined activated whole-cell bacterial extract and Derp1-specific immunotherapy in a patient with partial IgA deficiency and severe persistent rhinitis and moderate persistent asthma. The results over an 18-month period showed therapeutic efficacy characterized by a significant reduction in episodes of recurrent respiratory tract infections and asthma and allergic rhinitis control.

## 2. Case Presentation

The current case was an 11-year-old girl with IgA deficiency (7 mg/dl) and history of recurrent ear infections (8 to 10 episodes per year), tonsillitis (6 to 8 episodes per year), and pneumonia (2 episodes last year). The patient attended the Department of Allergy and Clinical Immunology Department, School of Medical and Health Sciences—SUPREMA, Juiz de Fora, Minas Gerais MG, Brazil. Clinical history showed the coexistence of persistent severe allergic rhinitis and moderate asthma. No history of skin allergy was noted. The prick test was performed to house dust mite, pollens, and fungus. Positive reactivity was observed (6 mm diameter) for *Dermatophagoides pteronyssinus.* Facial computed tomography showed partial opacification of the maxillary sinuses.

Treatment with amoxicillin + clavulanate (14 days) and prednisolone 40 mg (7 days) was started at the first visit. The use of nasal corticosteroids and inhaled corticosteroids + long-acting bronchodilator (LABA) was started for rhinitis and asthma control. Environmental control measures were also targeted.

At the second visit, antibiotic prophylaxis with sulfamethoxazole + trimethoprim (half the therapeutic dose) every other day was established after completing the initial antibiotic therapy cycle, and this therapeutic regimen was used for 3 months. During the same visit, the use of SLIT with Der p1 (IPI ASAC Brasil) was started with increasing daily doses for 4 months (induction phase) until reaching the maintenance dose (12 µg Derp1*/*month). Concomitantly, SLIT with inactivated whole-cell bacterial extract (IPI ASAC Brasil) was established using separate flasks and formulations, with fixed daily doses of 4 × 10^8^ colony-forming units (CFU) of inactivated bacteria by day. Inactivated whole-cell extract consisting of the following bacteria: *Streptococcus pneumoniae*, *Klebsiella pneumoniae*, *Moraxella* (*Branhamella*) *catarrhalis*, *Staphylococcus aureus*, *Haemophilus influenza*, *Streptococcus gordonii* (*S. mitis*), and *Streptococcus pyogenes*. The concentration used was 2 × 10^9^ CFU/mL.

Clinical monitoring was performed monthly during the first 4 months. The doses of inhaled corticosteroids + LABA and nasal corticosteroids were periodically reduced, and these drugs were discontinued after 6 and 3 months of treatment, respectively. The patient was evaluated every 2 months beginning from month 5. During the first 8 months after initiating treatment, the absence of episodes of respiratory infections of the upper and lower airways requiring the use of antibiotics was noted. During month 9, the patient experienced one episode of bacterial tonsillitis, which was treated with azithromycin combined with prednisolone with satisfactory results.

One year after treatment initiation, IgA was 3-fold relative to the initial value reaching 22 mg/dL. Currently, 18 months after the first visit, the patient has no history of episodes of bacterial respiratory infections, in addition to controlled allergic rhinitis and asthma. Cases of respiratory symptoms suggestive of viral infection were occasionally reported during clinical monitoring, and these episodes were controlled through symptom relief treatments.

## 3. Discussion

Recurrent respiratory infections are a public health problem observed in all ages. Ear infections, tonsillitis, sinusitis, and infections of the lower airways compromise both immunodeficient and immunocompetent individuals. *In situ* immune response deficiency in the respiratory tract mucosa is the key point that explains the presence of recurrent episodes of respiratory infections in individuals without systemic changes of the innate and/or acquired immune response [5–7]. As an aggravating factor, patients with asthma and allergic rhinitis may have a higher incidence of respiratory infections due to changes in the epithelial barrier of the respiratory mucosa and the presence of allergic inflammatory processes which upset the balance of local immunoregulatory mechanisms. Epithelial cells of the respiratory mucosa are immunologically active physical barriers interacting with innate and/or acquired immune response cells through cytokine and chemokine production [8, 9]. In patients with selective serum IgA deficiency, the commonly observed interaction with respiratory allergy produces an environment conducive to the development of recurrent respiratory infections.

Several clinical studies have demonstrated the efficacy of immunostimulation with bacterial extracts in the prophylaxis of recurrent infections of the upper and lower respiratory tract [5, 10–15]. These studies were performed in children and adults, demonstrating an excellent safety standard. However, the efficacy was variable, depending on the type of bacterial extract and administration route. The composition, dose, and administration route of bacterial extracts available in the market are highly heterogeneous. The extracts may have soluble proteins, particulate antigens, fragmented bodies, bacterial lysates, soluble protein mixtures, or inactivated whole-cell bacterial bodies. They may be administered subcutaneously, sublingually, or orally as capsules or tablets. Data suggest that inactivated whole-cell extracts administered at high concentrations produce consistent clinical results, preventing the development of recurrent respiratory infections [11, 16]. Therefore, inactivated whole-cell bacterial extracts formulated for use by doctors have a higher antigen concentration, thus increasing the chance of the efficient immunomodulation.

Capetti et al. demonstrated that four years of immunization with an immunostimulant preparation containing lysates of *Hemophilus influenzae*, *Streptococcus pneumoniae*, *Klebsiella pneumoniae*, *Klebsiella ozaenae*, *Staphylococcus aureus*, *Streptococcus pyogenes*, *Streptococcus viridans*, and *Neisseria catarrhalis* prevented respiratory infections in HIV+ patients [17].

In the present study, we used inactivated whole-cell extract consisting of the following bacteria: *Streptococcus pneumoniae*, *Klebsiella pneumoniae*, *Moraxella* (*Branhamella*) *catarrhalis*, *Staphylococcus aureus*, *Haemophilus influenza*, *Streptococcus gordonii* (*S. mitis*), and *Streptococcus pyogenes*. The concentration used was 2 × 10^9^ CFU/mL. Therefore, the total load of 12 × 10^9^ was administered in a 6 ml flask. Initially, in the first 4 months, the applications were daily, and the total load of 12 × 10^9^ CFU/mL inactivated bacteria was cumulatively administered for a 30-day period. Our results suggest that the long-term therapeutic regimen with a high cumulative total load of total body extract of inactivated bacteria, similar to that proposed for specific allergen immunotherapy, may be an effective strategy to promote prophylaxis against respiratory tract infections in selective IgA deficiency.

The mechanism of action for specific immunotherapy with bacterial extract is not yet fully understood. Several studies suggest that stimulation of dendritic cells by bacterial extract-derived antigens promotes T lymphocyte and phagocyte activation. These dendritic cells migrate to locoregional lymph nodes and present specific antigens to T lymphocytes. During antigen presentation, dendritic cells increase the expression of costimulatory molecules CD80, CD86, and CD83 and produce several cytokines, which modulate T lymphocyte activation and determine Th1 and Th2 polarization [5, 7]. In 2013, De Benedetto and Sevieri [13] proposed a consistent theory to explain the mechanism of action of lyophilized bacterial extracts administered orally as pills. Peyer's patch dendritic cells in the intestinal mucosa present specific bacterial extract antigens to TCD4+ lymphocytes, which initiates activation of specific T and B lymphocyte clones. These activated cells reach mesenteric lymph nodes and subsequently the respiratory tract mucosa, where they stimulate the innate and acquired immune response. Here they promote several events, including an increased cellular immune response specific to bacterial antigens present in the administered bacterial extract and increased secretory IgA synthesis. In patients with chronic obstructive pulmonary disease (COPD), the subcutaneous administration of lyophilized bacterial extract induced an increase in the serum levels of C-reactive protein and interleukin 6 (IL-6) with concomitant reduction of interleukin 8 (IL-8). Moreover, there was a decrease in respiratory infection episodes among patients with COPD [16].

The immunotherapy practice guidelines state that “Immunotherapy can be considered in patients with immunodeficiency and autoimmune disorders” [18, 19]. Sublingual immunotherapy (SLIT) appears to be better tolerated than subcutaneous immunotherapy (SCIT). The majority of SLIT adverse events are local reactions (e.g., oromucosal pruritus) that occur during the beginning of treatment and resolve within a few days or weeks without any medical intervention.

SLIT with *Dermatophagoides pteronyssinus* combined with bacterial extract in children promoted increased salivary IgA levels associated with decreased allergen-specific serum IgE levels [20]. Allergen-specific immunotherapy has the ability to promote control of allergic processes and decrease the number of episodes of viral and bacterial respiratory infections in atopic individuals [19, 21, 22]. In the present case report, when the patient showed positive skin-test reactivity to the allergen, we chose to initiate allergen-specific immunotherapy concomitantly with immunotherapy using inactivated whole-cell bacterial extract. We observed a positive outcome, preventing episodes of both allergy and respiratory infections.

The difficulty of performing double-blind, placebo-controlled clinical trials in this specific population of patients with partial IgA deficiency precludes the examination of mechanisms of action of allergen-specific immunotherapy with bacterial extracts in these cases of immunodeficiency. However, published clinical trials show the efficacy of bacterial extracts, regardless of combination with allergen-specific immunotherapy, in the prophylaxis of recurrent respiratory infections. We believe that this case report may contribute to expanding the therapeutic perspective of individuals with partial selective serum IgA deficiency.

In the present case report of a patient with partial IgA deficiency, immunotherapy with inactivated whole-cell bacterial extract concurrently with Der p1, clinically monitored for an 18-month period, promoted a significant decrease in the number of episodes of respiratory infections as well as allergic rhinitis and asthma control. A 3-fold increase in serum IgA levels compared to treatment initiation was also observed. We emphasize that separate flasks of inactivated whole-cell bacterial extract and Der p1 were used, thus enabling us to reach adequate therapeutic concentrations and avoiding any possibility of biochemical interactions between components of the different extracts. This case demonstrates the potential utility of the concurrent use of SLIT with inactivated whole-cell bacterial extract and Der p1 for successful control of allergy and infection in patients with selective IgA deficiency.
